# Onchocerciasis and trachoma control: what has changed in the past two decades?

**Published:** 2008-09

**Authors:** Daniel Etya'ale

**Affiliations:** Coordinator of VISION 2020 in Africa, Programme for the Prevention of Blindness, World Health Organization, 20 Avenue Appia, CH-1211 Geneva 27, Switzerland.

**Figure:**
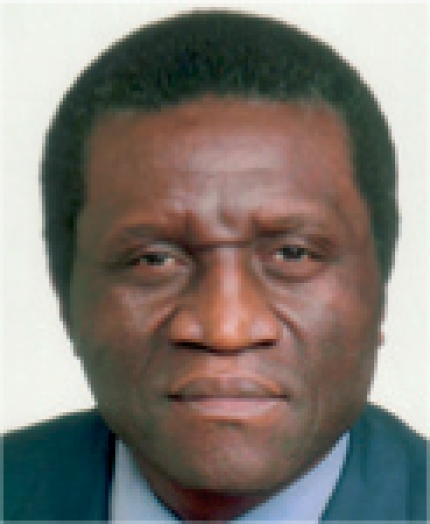


Trachoma and onchocerciasis are the two major infectious causes of blindness worldwide. Twenty years ago, the possibility of achieving worldwide and long-term control of these ancient scourges seemed remote and existing control programmes were deemed to have limited prospects. The picture is very different today: large-scale interventions to control both diseases are not only expanding, but control and even elimination are now being discussed as real achievable goals in a growing number of countries. As we will show in this mainly programmatic review, this is a remarkable achievement over only two decades!

## From slow and uncertain beginnings to large-scale efforts

### Onchocerciasis

Up until the late 1980s (see Table 1), the only established disease control activity was the Onchocerciasis Control Programme (OCP), a World Health Organization (WHO) programme jointly sponsored by the World Bank, the United Nations, and a coalition of over 20 donor countries and agencies. Set up in 1974 in seven, then 11, countries in West Africa, OCP was the first programme to demonstrate that control of onchocerciasis as a public health problem was indeed possible. However, many thought this concerned only 11 countries. Moreover, neither the strategy (vector control), nor the tools developed by, and for, OCP operations (weekly aerial applications of larvicide, ongoing monitoring of community microfilarial loads and flies' infectivity, etc.), nor the financial support available at the time, would lend themselves to further extension of control activities to the other endemic countries (19 in Africa and six in Central and Latin America).^1^

**Table 1:** A chronological outline of the development of onchocerciasis control programmes since 1974

Date	Key event
1974	•OCP is established in West Africa, using vector control as the sole strategy
1987	•Mectizan® Donation Programme is launched
1989–1990	•Large community-sscale trials show the benefit of Mectizan®
	•First NGDO-ssupported Mectizan® distribution projects, using mobile strategies
1992	•The NGDO Group for Mectizan® distribution is established within the WHO Prevention of Blindness Programme
	•OEPA is launched
1994	•REMO is developed to define priority areas for disease control
1995	•CDTI is recommended as a safe and cost-effective strategy for onchocerciasis control
	•APOC is launched
1999	•VISION 2020 is launched
2002	•OCP winds down its activities

**Figure:**
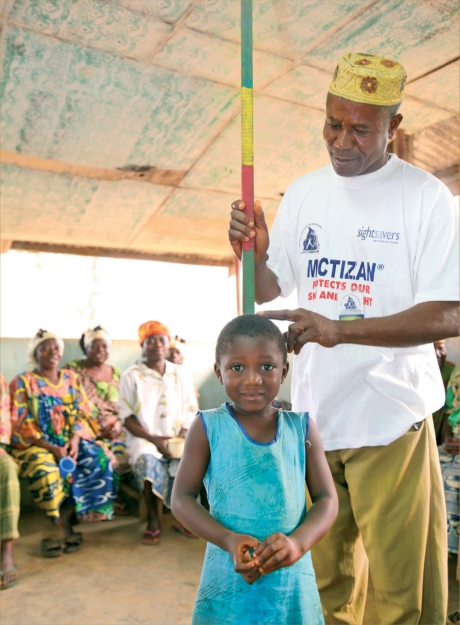
The donation of Mectizan® by Merck & Co from 1987 has had a unique impact on the worldwide fight against onchocerciasis

All this changed in 1987 with a first and historic donation of ivermectin (Mectizan®) by Merck & Co.^2^ For the first time, a safe and effective microfilaricide was not only available, but, as subsequent studies quickly established, could lend itself to mass treatments in high-risk, endemic communities. The wide and generous availability of Mectizan® also accelerated operational research activities and the development of new tools – for example Rapid Epidemiological Mapping of Onchocerciasis (REMO) was developed to precisely map all priority areas in each endemic country^3^ and it emerged that Community-Directed Treatment with Ivermectin (CDTI) was the most appropriate and cost-effective method for community-wide delivery of the new drug. CDTI was advocated when the African Programme for Onchocerciasis Control (APOC) was launched in 1995, and it remains the current strategy of all programmes today. In addition, CDTI is now being used to distribute other public health interventions such as vitamin A and bednets to prevent malaria.

Looking back, this historic and still unsurpassed donation of Mectizan®, “to as many as need it, for as long as needed,” is the one essential ingredient that has uniquely impacted nearly every major development in the worldwide fight against onchocerciasis since then. It has also inspired other major drug donation initiatives, such as that of albendazole by GlaxoSmithKline in 1998 for lymphatic filariasis, and that of azithromycin by Pfizer Inc. for trachoma the same year.

### Trachoma

Modern efforts to control trachoma date back to the early 1950s, with the establishment, through WHO's support, of national programmes in endemic countries in the Western Pacific, Asia, and the Middle East (see Table 2). These activities also included the assessment of the magnitude of blinding trachoma in these regions and the institution, where feasible, of operational research on treatment options. However, these efforts were rarely sustained. This was due partly to the lack of a simple tool to assess and grade trachoma and to the nearly insurmountable challenge of fostering community-wide compliance for a six-week treatment regimen of twice-daily application of a tetracycline ointment, in the absence of parallel measures to transform at the same time the environment in which trachoma thrives. As a result, trachoma attracted only marginal public interest until the mid-1990s.^4^

**Table 2:** A chronological outline of the development of trachoma control programmes since the mid-twentieth century

Date	Key event
1950s and 1960s	•Establishment of National Trachoma Control Programmes, mainly in endemic Asian, Middle Eastern, and Western Pacific countries
1987	•Introduction of the simplified grading scheme for trachoma
1996	•Introduction of the SAFE strategy
1997	•Launch of GET 2020
1998	•First donation of azithromycin and establishment of the International Trachoma Initiative
1999	•Launch of VISION 2020

The introduction of the simplified grading scheme for trachoma in 1987 and of the SAFE strategy (Surgery, Antibiotics, Facial cleanliness and Environmental changes) in 1996 by WHO^5^ represented crucial operational milestones in trachoma control. This led to three important developments, which provided the impetus needed to put this ancient disease back on the world map in the mid-1990s: (i) the establishment of the WHO GET 2020 alliance (Global Elimination of Blinding Trachoma by the Year 2020), providing the mandate and framework for trachoma control worldwide; (ii) the donation of azithromycin by Pfizer Inc. in 1997; and (iii) the subsequent establishment in 1998 of the International Trachoma Initiative (ITI), to manage this donation. This served as a catalyst to expand and accelerate ongoing control activities, a process further facilitated by the ease with which azithromycin can be administered even on a large scale (a single oral dose in most cases, as opposed to the six-week tetracycline regimen).

## Some major achievements to date

### Onchocerciasis

OCP wound down its activities in 2002, having achieved the prevention of 600,000 cases of blindness and protected a further 40 million people from ocular morbidity throughout large areas in West Africa. However, it was also agreed that in order to prevent recurrence of the disease and consolidate these important gains, distribution of Mectizan® must be continued, with high coverage, and robust surveillance systems established and maintained.

Elsewhere, control activities now cover nearly all known meso- and hyper-endemic areas around the world. APOC thus aims to protect some 92 million at-risk individuals from the deleterious effects of river blindness; currently more than half of them are under annual Mectizan® treatment.

Similarly, the Onchocerciasis Elimination Programme for the Americas (OEPA) has established, in all six endemic countries, effective national programmes in all 13 foci with a treatment coverage of at least 85% twice a year. Even more significantly in 2007, all eye lesions attributable to onchocerciasis had been eliminated in nine of these 13 foci.

### Trachoma

An increasing number of endemic countries are now receiving support for baseline surveys, national plan development, the implementation of the SAFE strategy, and the development and use of appropriate indicators for monitoring and evaluation purposes. Others, like Morocco, the first country to have completed its campaign for trachoma control in 2006, are now awaiting WHO certification for the elimination of blinding trachoma as a public health problem throughout the country. Other countries still (The Gambia, Ghana, Mauritania, and Viet Nam) are also well on track to completing their trachoma control campaigns by 2010.

## Future prospects

There is little doubt that, because of ongoing activities and the remarkable achievements to date, onchocerciasis and blinding trachoma may become the first major causes of needless blindness to achieve VISION 2020 objectives within the year 2020 endpoint.

APOC's operations are now scheduled to end by 2015. Current thinking and consensus is that, by then, the primary objective of the Programme, i.e. to establish sustainable national onchocerciasis control activities in all endemic countries, may not be achieved everywhere. This is mainly because programme implementation has been significantly slowed down in war-torn countries, for obvious reasons, and in Central Africa where co-endemicity with Loa Loa and the risk of severe Central Nervous System complications has required extreme care and close medical supervision in the distribution of Mectizan®.

It is therefore imperative to ensure that all residual activities, including post-treatment surveillance, will have the financial and other logistic support needed for their completion or, failing that, for their integration into viable national health care systems.

Regarding trachoma control, the coming years should see a further expansion of the SAFE strategy and an increasing number of endemic countries with fully developed national plans. Hopefully, both developments will be matched with a similar increase in financial resources available at country level.

**Figure:**
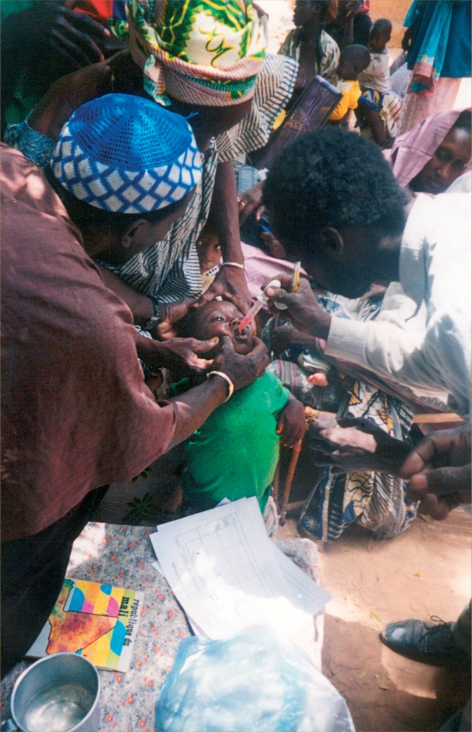
The SAFE strategy, introduced in 1996, was one of the events that put the fight against trachoma back on the map

## Conclusion

Despite being two very different diseases, onchocerciasis and trachoma have a lot in common. Both are diseases of poverty, often affecting not just the poorest among the poor, but also the most difficult to reach in communities often described as “at the end of the road.” The challenges that they pose for their control are also quite similar, in that successful control of both diseases requires far more than an effective strategy or a freely available drug. Just as essential is highly coordinated work between all players involved, from donors and researchers, to people working in the field, not forgetting affected communities themselves.

Trachoma control presents us with an additional (and major) operational challenge, in that the success of the SAFE strategy requires a close and essential collaboration with non-medical experts for the implementation of its ‘F’ and ‘E’ components. Failing to fully implement all of its four components will mean running the risk of reducing the SAFE strategy into a purely medical effort that, most agree, is not likely to achieve optimal success – if at all – in our fight against blinding trachoma.

**Ten reasons for success**

**The rapid progress and success achieved so far by onchocerciasis and trachoma control programmes is due to a combination of many contributing factors. These include:**

1. **Drug donations by pharmaceutical companies**: the historic, generous and timeless donation by Merck & Co. of Mectizan® for onchocerciasis control activities, and Pfizer Inc.'s donation of azithromycin for trachoma control in a number of affected countries.
2. **The development of cost-effective, rapid assessment methodologies** which facilitated the mapping of the disease, such as: REMO (Rapid Epidemiological Mapping of Onchocerciasis) and REA (Rapid Epidemiological Assessment) for onchocerciasis, or the simplified grading scheme for trachoma, which facilitated the identification of priority areas.
3. **Development of country databases for planning, monitoring and evaluation**: extensive, user-friendly, interactive databases, with detailed information on all endemic communities, target populations, nearby schools and health facilities, roads, etc.
4. **Regular maintenance and updating of these databases in each country** by well-trained and capable local teams.
5. **Secure and predictable financing over many years** ensuring that planned activities will indeed be implemented: 27 years for former OCP; ongoing since 1992 and 1995, respectively, for OEPA and APOC.
6. **The establishment of a solid public-private partnership and the meeting once a year of all stakeholders** to review past and future programme activities and more importantly, to agree on next year's budget for the programme.
7. **The existence in each endemic country of well-structured and truly functional national Onchocerciasis Task Forces** (NOTF), in which all stakeholders (programme managers, NGDOs, Ministry of Health officials, researchers) meet regularly to plan, implement, monitor, and evaluate together all ongoing control activities.
8. **The promotion, generous support and regular use of operational research** on all core aspects of programme implementation and its ‘feedback’ into ongoing operations.
9. **A flexible and adaptive approach to mass distribution**: e.g. mass distribution of Mectizan® has evolved from mobile strategies (very expensive and hardly sustainable) to community-directed treatment with ivermectin (high community ownership, very cost effective, and more likely to be sustainable).
10. **Active involvement of target communities**: the prime beneficiaries of the programme, i.e. affected communities, are actively involved at all stages of programme implementation (planning, community mobilisation, motivation of distributors, implementation, supervision, monitoring – including self-monitoring and supervision). A sharp contrast to what still prevails in many health intervention programmes, where targeted populations have no other role to play except that of passive but grateful participants of well-designed and scientifically sound programmes developed on their behalf and for their benefit.

**A challenge for the future: moving out of our comfort zone**

Past experience has consistently shown that medical personnel tend to implement only the S (Surgery) and A (Antibiotic) components of the SAFE strategy. Reasons for this include:

- The ‘S’ and ‘A’ components are the ones that health workers are most comfortable with or have skills for.
- The Ministry of Health rarely has the skills, expertise, and resources needed for the effective implementation of the ‘F’ and ‘E’ components.
- The ‘F’ and ‘E’ components require input from, and a close working relationship with, experts in the fields of education, community development, water, sanitation, and hygiene.
- To this date, in many endemic countries, trachoma and public health experts have shown limited willingness for, and experience with, such a close and synergistic collaboration with non-medical experts.

While there is no doubt that the ‘S’ and ‘A’ components remain important and urgent, the natural history of trachoma, in those parts of the world where it was once endemic, is also there to remind us that the disappearance of the disease had little to do with effective medical intervention (nonexistent at the time), but everything to do with improved socioeconomic living conditions, better sanitation, easy access to water, etc.

Seen in that light, trachoma control presents to medical professionals a far greater challenge than any other cause of avoidable blindness. The imperative to make it succeed leaves us with no other option but to move out of our comfort zone and to proactively seek and reach out to other players, whose contribution to our global success may turn out at the end to be the ‘essential one’.

A humbling challenge indeed for some, but also a unique opportunity to develop tomorrow's leaders, many of whom will be operating more and more (and not less) in resource-constrained environments, involved in more and more complex interventions such as, the Millennium Development Goals or the co-implementation of Neglected Tropical Diseases.
